# The Stability of Hybrid Perovskites with UiO-66 Metal–Organic Framework Additives with Heat, Light, and Humidity

**DOI:** 10.3390/nano12234349

**Published:** 2022-12-06

**Authors:** Ivan S. Zhidkov, Ming-Hsuan Yu, Andrey I. Kukharenko, Po-Chun Han, Seif O. Cholakh, Wen-Yueh Yu, Kevin C.-W. Wu, Chu-Chen Chueh, Ernst Z. Kurmaev

**Affiliations:** 1 Institute of Physics and Technology, Ural Federal University, Mira St. 19, 620002 Yekaterinburg, Russia; 2 M.N. Mikheev Institute of Metal Physics of Ural Branch of Russian Academy of Sciences, 620108 Yekaterinburg, Russia; 3 Department of Chemical Engineering, National Taiwan University, Taipei 10617, Taiwan; 4 Program of Green Materials and Precision Devices, International Graduate Program of Molecular Science and Technology, Center of Atomic Initiative for New Materials, National Taiwan University, Taipei 10617, Taiwan; 5 Advanced Research Center for Green Materials Science and Technology, National Taiwan University, Taipei 10617, Taiwan

**Keywords:** stability, hybrid perovskites, XPS, UiO-66, metal–organic frameworks

## Abstract

This study is devoted to investigating the stability of metal–organic framework (MOF)-hybrid perovskites consisting of CH_3_NH_3_PbI_3_ (MAPbI_3_) and UiO-66 without a functional group and UiO-66 with different COOH, NH_2_,and F functional groups under external influences including heat, light, and humidity. By conducting crystallinity, optical, and X-ray photoelectron spectra (XPS) measurements after long-term aging, all of the prepared MAPbI3@UiO-66 nanocomposites (with pristine UiO-66 or UiO-66 with additional functional groups) were stable to light soaking and a relative humidity (RH) of 50%. Moreover, the UiO-66 and UiO-66-(F)_4_ hybrid perovskite films possessed a higher heat tolerance than the other two UiO-66 with the additional functional groups of NH_2_ and COOH. Tthe MAPbI3@UiO-66-(F)_4_ delivered the highest stability and improved optical properties after aging. This study provides a deeper understanding of the impact of the structure of hybrid MOFs on the stability of the composite films.

## 1. Introduction

Despite the dramatic increase in the efficiency of hybrid perovskite solar cells (PVSCs) from 3.8% to 25.7% achieved recently, their commercialization has been significantly delayed due to the problems associated with their long-term stability [1,2,3,4,5,6,7]. As a result of the use of the generally accepted technology for their manufacture based on solution-based processes, the obtained films with typical ionic crystal structures usually have many defects and grain boundaries, which destabilizes the operation of the devices. As a consequence, the hybrid perovskites are prone to degradation when exposed to moisture, oxygen, heat, and light [8,9,10]. To increase the stability of perovskite materials to these external influences, compositional engineering is often used to replace the unstable organic cations with their inorganic counterparts [11,12,13], or functional additives are used to regulate the crystallinity [14,15]. Methylammonium lead triiodide (MAPbI_3_), which has received great attention and has made comprehensive progress in stabilizing perovskite films and establishing a degradation mechanism, has been studied for fabricating efficient, stable, and scalable perovskite solar cells [16,17,18,19,20]. Very recently, the use of a metal–organic framework (MOF) as a platform for hosting (encapsulation) hybrid perovskites has attracted increasing attention to improve their photochemical and thermal stability [21,22,23,24,25]. Using simple synthetic procedures, it is possible to convert perovskite nanoparticle (NP)@MOF suspensions into microporous films/scaffolds using a centrifugation process or by mixing them with other precursors involved in the manufacture of photovoltaic devices [26,27].

In this study, the resistance to light, heat, and high relative humidity (RH) of a series of MAPbI_3_@UiO-66 nanocomposites with different functional groups was studied. Lewis base groups, such as carboxyl (COOH), amine (NH_2_), and hydrophobic fluorine (F) groups, have been reported to provide good passivation effect and humidity stability and were chosen as functional groups for the design of UiO-66 MOFs [28,29,30]. Through the analyses of the crystallinity and optical properties and X-ray photoelectron spectroscopy (XPS), we obtained a deeper understanding of the impact of the structure of the hybrid MOF on the stability of the composite film. The hybrid UiO-66-based MOFs were shown to greatly enhance the moisture stability and UV-light irradiation stability of the derived composite films regardless of the structures of the constituent organic ligands. MAPbI_3_@UiO-66 and MAPbI_3_@UiO-66-(F)_4_ were shown to yield greater thermal stability than MAPbI_3_@UiO-66-NH_2_ and MAPbI_3_@UiO-66-(COOH)_2_ as a result of their higher affinity with the perovskite crystals [26,31].

## 2. Experimental Section

### 2.1. Materials and Sample Preparation

Lead iodide (PbI_2_, 99.99%) and methylammonium iodide (MAI, >99.99%) were purchased from TCI chemicals and Greatcell Solar Materials, respectively. The organic solvents for film preparation were purchased from Sigma Aldrich (St. Louis, MO, USA) and used without purification. Equimolar PbI_2_ and MAI were dissolved in a mixed solvent of dimethylformamide (DMF) and dimethyl sulfoxide (DMSO) with a volume ratio of 8.9:1.1 and with a concentration of 1.5 M to form the MAPbI_3_ perovskite precursor solution [32]. The MAPbI_3_@MOF nanocomposites were prepared on an SiO_2_ substrate according to a similar method previously reported, where the blending of the MOF amount was set to be 0.03 wt% in order to maintain the high PCE of the derived solar cell devices [26]. The precursor solutions for the MAPbI_3_@MOF nanocomposites were prepared as follows. All the MOF suspension solutions were pre-dispersed in DMF with a concentration of 1 mg/mL. Next, the MAPbI_3_ perovskite precursor solution and the MOF suspension solutions were mixed with a volume ratio of 4:1 to form the MAPbI_3_@UiO-66 precursor solutions with ~0.03 wt% MOF. The SiO_2_ substrates were first treated with air plasma for 10 min to remove the unwanted residues and to increase the surface’s hydrophilicity. The films of the MAPbI_3_@UiO66 nanocomposites were prepared using an antisolvent method. In brief, the precursor solutions were spin-coated onto the SiO_2_ substrate at 4000 rpm for 20 s, and diethyl ether (DEE) was dripped onto the substrate after 7 s of the spin-coating process, followed by thermal annealing at 60 °C for 1 min and 100 °C for 30 min. The blended MOFs were supposed to distribute over the perovskite grain boundary rather than embed in the grain according to the XRD results reported in our previous work [26]. The reference MAPbI_3_ sample was also prepared for a fair comparison. Hereafter, the control MAPbI_3_ sample is designated as “pristine”, and the MAPbI_3_@UiO-66 nanocomposites are denoted according to the types of hybrid UiO-66 as MAPbI_3_@UiO-66, MAPbI_3_@UiO-66-NH_2_, MAPbI_3_@UiO-66-(COOH)_2_, and MAPbI_3_@UiO-66-(F)_4_.

### 2.2. Characterization

The field-emission gun scanning electron microscope (FEG-SEM) images were taken using a Nova NanoSEM 230 (FEI, Hillsboro, OR, USA). The XRD patterns were characterized using a Rigaku SmartLab SE (Rigaku, Tokyo, Japan). The ultraviolet visible absorption (UV-Vis) and photoluminescence (PL) spectra were recorded using a Hitachi U-4100 UV–visible spectrophotometer (Hitachi, Tokyo, Japan) and a Horiba Fluorolog-3 spectrometer system (Horiba, Kyoto, Japan), respectively. X-ray photoelectron spectroscopy (XPS) was used to measure the core level spectra of the MAPbI3@UiO-66 nanocomposites with the assistance of a PHI XPS 5000 VersaProbe spectrometer (ULVAC-Physical Electronics, Chanhassen, MN, USA) equipped with a spherical quartz monochromator and an energy analyzer working in the range of binding energies from 0 to 1500 eV. The energy resolution (ΔE) was ≤0.5 eV. Finally, the XPS spectra were processed using PHI MultiPak 9.9.0.8 software (ULVAC-Physical Electronics, Chanhassen, MN, USA).

## 3. Results and Discussion

**Figure 1:**
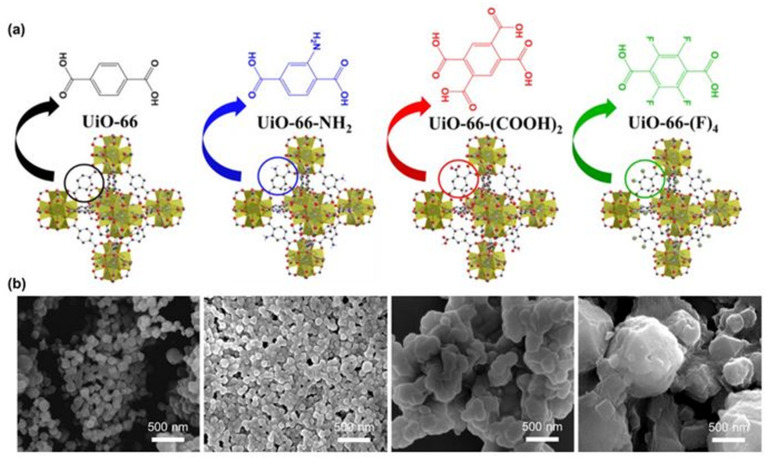
(**a**) The structures of the UiO-66-based MOFs with different functional groups, including UiO-66, UiO-66-NH_2_, UiO-66-(COOH)_2_, and UiO-66-(F)_4_ and (**b**) the corresponding SEM images of these synthesized MOFs.

**Figure 2:**
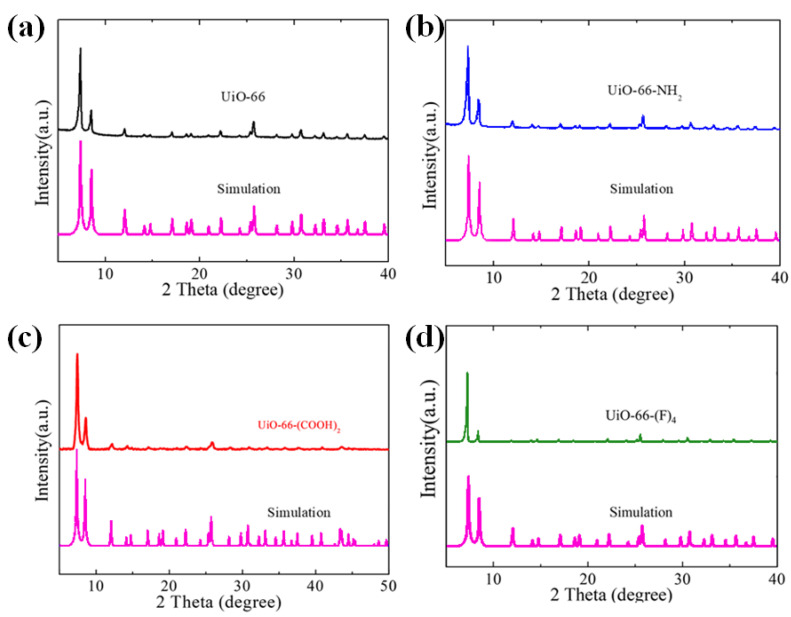
The XRD spectra of the synthesized MOFs: (**a**) UiO-66, (**b**) UiO-66-NH_2_, (**c**) UiO-66-(COOH)_2_, and (**d**) UiO-66-(F)_4_.

As demonstrated in our previous work and other recent studies [21,22,23,26], blending a small amount of MOFs into the perovskite layers can improve the film quality and stability. The MOFs tend to distribute at the perovskite grain boundaries, which leads to lower defect concentrations and helps to stabilize the perovskite crystal with its high chemical and thermal resistance [26,31]. In this context, we systematically investigated the thermal/photo/moisture stability of a series of MAPbI_3_@UiO-66 nanocomposites using a pristine UiO-66 without any functional group (MAPbI_3_@UiO-66) and UiO-66 with different functional groups, including NH_2_, COOH, and F (MAPbI_3_@UiO-66-NH_2_, MAPbI_3_@UiO-66-(COOH)_2_, and MAPbI_3_@UiO-66-(F)_4_), and compared these to the pristine MAPbI_3_ film (Figure 1a) [33]. The syntheses of the UiO-66-based MOFs, UiO-66, UiO-66-NH_2_, UiO-66-(COOH)_2_, and UiO-66-(F)_4_ adopted the methods reported in the previous work [33]. Figure 1b presents the scanning electron microscope (SEM) images of the prepared MOFs in particle form, and their corresponding X-ray diffraction (XRD) patterns are displayed in Figure 2, which confirmed the successful preparation of these targeted MOFs. We were interested in understanding the structural influence of this emerging additive on the resultant stability against heat, light, and moisture. Accordingly, we specifically set up three aging conditions for the experiments: (i) exposure to air with an RH of ~50% (RH 50%) to study the moisture stability, (ii) UV-light soaking with a power of ~20 mW/cm^2^ to study the photostability, and (iii) continuous heating at 85 °C on a hot plate to investigate the thermal stability. Note that, for the humidity stability experiment, the samples were stored in the dark, and for the other two stability experiments, testing was performed in a nitrogen atmosphere to limit the influences from other factors.

**Figure 3:**
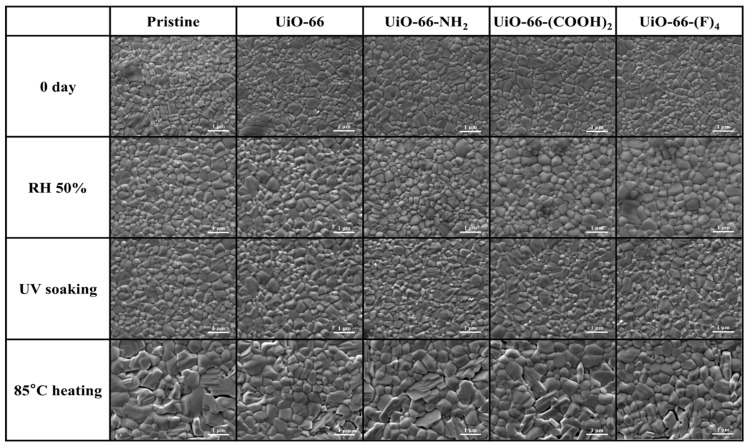
The SEM images of the pristine MAPbI_3_ and MAPbI_3_@UiO-66 nanocomposite samples before and after humidity, UV-light, and heat aging for two weeks.

**Figure 4:**
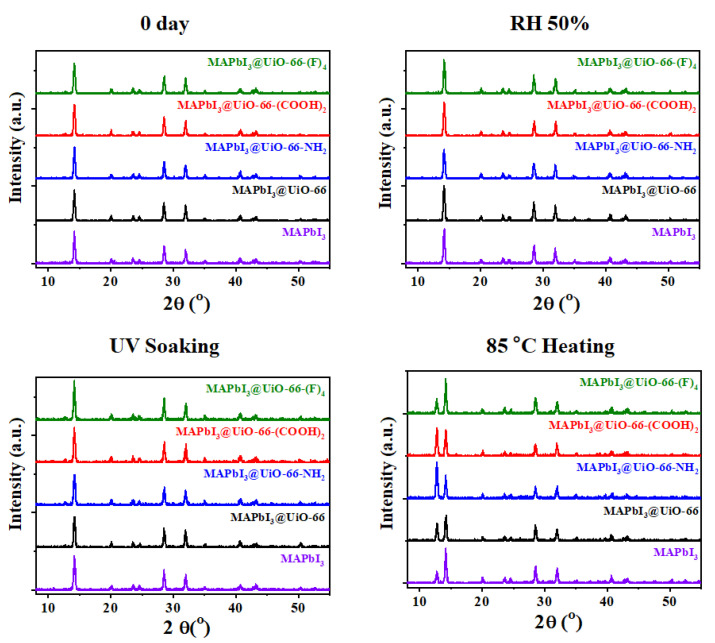
The XRD spectra of the pristine MAPbI_3_ and MAPbI_3_@UiO-66 nanocomposite samples before and after humidity, UV-light, and heat aging for two weeks.

**Figure 5:**
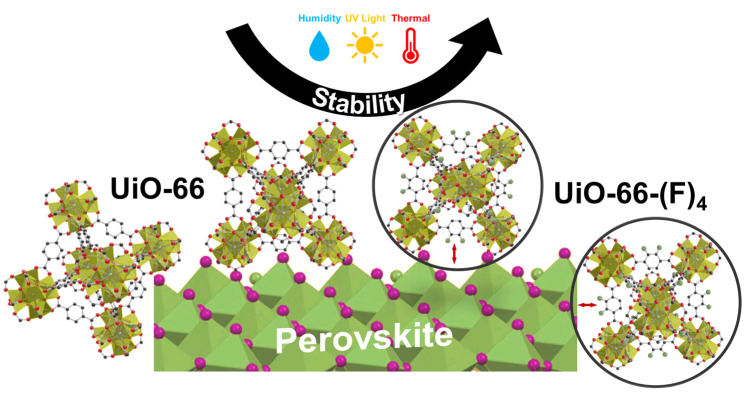
Schematic of the interaction of perovskite/UiO-66 and perovskite/UiO-66-(F)_4_.

It is well known that PbI_2_ is one of the major residual products of the common perovskites (such as MAPbI_3_ and FAPbI_3_) after degradation. Therefore, we can easily identify the degradation of perovskite by crystallinity and optical analyses, based on the distinctive features of the lattice structure and yellowish appearance of PbI_2_ [34,35]. Figure 3 presents the field emission gun scanning electron microscope (FEG-SEM) images of the pristine MAPbI_3_ and MAPbI_3_@UiO-66 nanocomposite samples before and after humidity, UV-light, and heat exposure for two weeks, and their corresponding XRD patterns are displayed in Figure 4. Overall, there was not much difference before and after aging for the UV-irradiated and humidity-aged samples, in contrast to the heat-exposure samples. However, if we examine the samples closely, we see that, after aging by UV irradiation or by humidity exposure for two weeks, the perovskite grains of these samples became denser and smoother. Moreover, the full width at half maximum (FWHM) from the corresponding XRD spectra became slightly smaller. These changes indicate that the perovskite grains underwent certain crystal rearrangements that resulted in a higher crystallinity. As reported in several previous studies [36,37], the perovskite recrystallization may occur over a couple of days in mild environments such as nitrogen or dry air. Despite this, excessive exposure to oxygen, moisture, or other stimuli will reduce the crystallinity-enhancing effects and engender degradation. As evidenced by the above results, the thermally driven decomposition greatly dominated the perovskite degradation despite being stored in a nitrogen environment. As presented in Figure 3, conspicuous cracks and hexagonal PbI_2_ appeared in the samples under a constant heat stress. The MAPbI3@UiO-66 and MAPbI3@UiO-66-(F)_4_ samples displayed relatively intact morphology along with lower PbI_2_ signals (at ~12.7°, Figure 4), compared to the other samples. We attributed this observed increase in thermal stability to the relatively higher structural affinity of both hybrid MOFs toward the perovskite crystals, which led to a lower pinhole formation. Furthermore, the halogen bonding of the fluorine group may promote additional perovskite–MOF interactions (Figure 5) without interrupting the perovskite grain connection. The above advantages helped to stabilize the surface defects and reduce the degradation rate [26,31].

**Figure 6:**
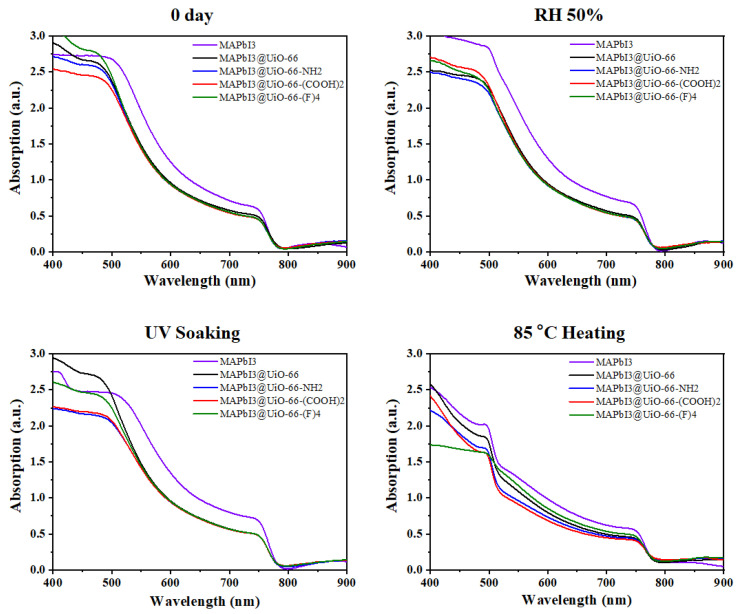
The UV-Vis spectra of the pristine MAPbI_3_ and MAPbI_3_@UiO-66 nanocomposite samples before and after humidity, UV-light, and heat aging for two weeks.

We next analyzed the UV-Vis absorption of these samples before and after aging. Specifically, we recorded and traced the characteristics of the band-edge absorption, PL intensity, and its peak shift to examine the difference caused by aging [38,39]. Figure 6 displays the UV-Vis spectra of the pristine MAPbI_3_ and MAPbI_3_@UiO-66 nanocomposite samples before and after humidity, UV-light, and heat exposure for two weeks. Consistent with the aforementioned results, the samples retained the unique dark brown color of perovskite without showing a distinguishable difference, except for the heat-aged samples. The UV-irradiated and humidity-aged samples possessed sharper absorption edges, which represented a lower band-tail recombination. This can be attributed to the age-induced recrystallization, as discussed earlier [36]. On the other hand, the heat-aged MAPbI_3_@UiO-66 and MAPbI_3_@UiO-66-(F)_4_ samples similarly exhibited a lower reduction in absorption compared to the freshly prepared samples than the others, revealing a superior thermal stability.

**Figure 7:**
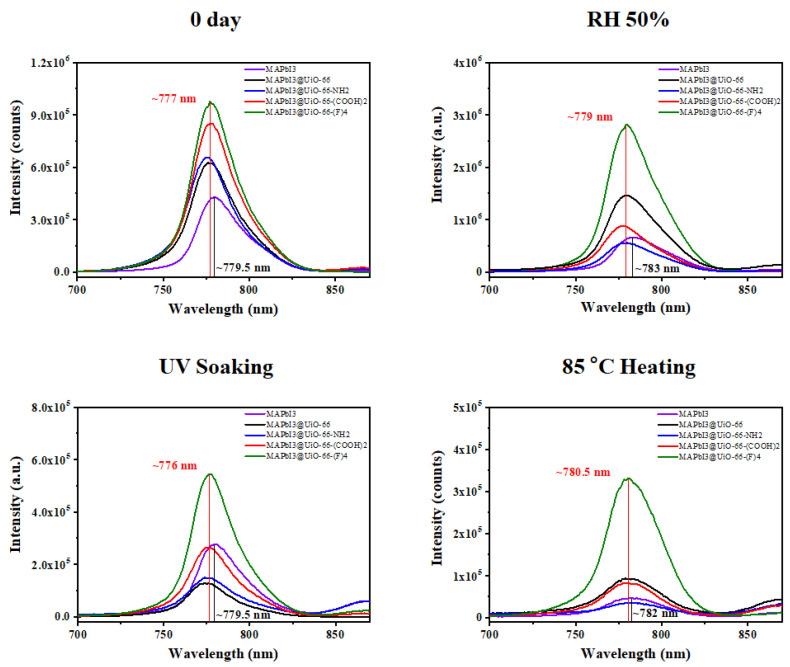
The PL spectra of the pristine MAPbI_3_ and MAPbI_3_@UiO-66 nanocomposite samples before and after humidity, UV-light, and heat aging for two weeks.

Figure 7 displays the PL spectra of these samples before and after the humidity, UV-light, and heat exposure for two weeks. As can be seen, all of the aged MAPbI_3_@UiO-66 nanocomposite samples delivered blue-shifted PL peaks compared to the pristine MAPbI_3_, along with increased peak intensity. This showed that the hybrid MOFs could potentially passivate the perovskite grain boundary or the associated defects [38]. The humidity-aged samples delivered red-shifted peaks compared to the fresh samples, accompanied with an enhanced PL intensity and enlarged FWHM values. We speculate that the increased PL intensity was related to the defect passivation exerted by water and oxygen, which resulted in a reduced non-radiative recombination. However, excessive water and oxygen still form intermediate products with perovskite and finally trigger the degradation, as evidenced by the red-shifted PL peaks and the enlarged FWHM values [37,40]. On the other hand, the UV-irradiated samples generally expressed slightly lower PL intensity than the fresh samples, without showing any peak shifts. This reveals that these samples were relatively stable under low-intensity light irradiation when stored in a nitrogen environment. Finally, as shown, the heat-treated samples had the lowest PL intensity along with the most red-shifted PL peak and the largest peak broadening among all the aging conditions. This reveals that these aged perovskite films possessed the highest amount of surface defects (Figure 3) and the non-perovskite phase, which caused serious degradation (Figure 4). However, the MAPbI_3_@UiO-66-(F)_4_ film had the highest PL intensity, along with a distinctive blue-shifted peak. This indicates that the hybrid UiO-66-(F)_4_ stabilized the associated perovskite interfaces in a more effective manner, with the halogen bonding or hydrogen bonding of the fluorine group with perovskite, than the other nanocomposites. Based on the above results, we can conclude that the hybrid MOFs potentially passivated the perovskite grain boundaries and provided grain-locking effects to present higher resistance without interfering with the perovskite crystal growth [30]. Furthermore, MAPbI_3_@UiO-66-(F)_4_ particularly afforded the best stability among the hybrid MOFs, benefitting from its relatively higher affinity with the perovskite crystals [26,31]. These results clearly indicate the distinct influences of the functional groups of MOFs on stabilizing the perovskite interface. In rationally fine-tuning the functional group, the stability of the hybrid MAPbI_3_@UiO-66 film against heat, light, and humidity was further enhanced.

**Figure 8:**
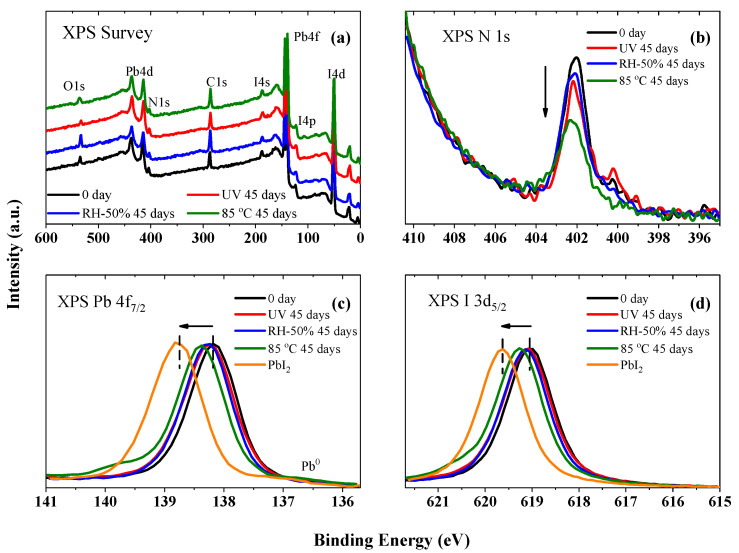
(**a**) XPS survey and (**b**) N 1s, (**c**) Pb 4f_7/2_, and (**d**) I 3d_5/2_ spectra of the pristine sample before and after light soaking, heat stress, and relative humidity treatment for 45 days.

To gain further insight into the compositional changes of these MAPbI_3_@MOF nanocomposites after different aging conditions, XPS measurements were conducted. The results of the XPS survey spectra of the pristine MAPbI_3_ sample are presented in Figure 8. There were no uncontrolled impurities on the surface of the sample under study, indicating a high quality of the prepared perovskite film. To analyze the local atomic and electronic structures of the pristine MAPbI_3_ and its changes after exposure to light, constant heat, and humidity, the high-energy resolved XPS spectra of the core levels (XPS N 1s, I 3d, and Pb 4f) were studied (see Figure 8b–d). According to these data, which were in good agreement with our preliminary XPS measurements [41], the relative intensity of the XPS N 1s-spectra perovskites, normalized to the XPS Pb 4d-line intensity (Figure 8), decreased under the external influences (especially strong under a constant heat stress), which indicated the breaking of the C-N bonds and the partial decomposition of the organic cation. In the I 3d (Figure 8c) and Pb 4f (Figure 8d) spectra, a high-energy shift was recorded in the spectra of the samples treated with light, heat, and humidity in the direction of the spectrum of the reference PbI_2_ (Table 1). This indicated an increase in the precipitation of the PbI_2_ phase as a decomposition product of the MAPbI_3_ perovskite [42]. These data showed that the initial samples of MAPbI_3_ perovskite exhibited long-term photochemical and thermal instability and also partially decomposed when exposed to a humid environment.

**Figure 9:**
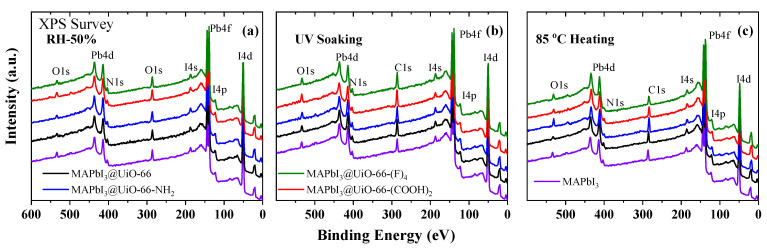
XPS survey spectra of the MAPbI_3_@UiO-66 nanocomposites before and after storage at (**a**) RH 50%, (**b**) UV light, and (**c**) annealing at 85 °C for 45 days.

Our next step was to conduct similar research on the MAPbI_3_@UiO-66 nanocomposites. First of all, we measured their XPS survey spectra as shown in Figure 9a–c. Similar to the pristine MAPbI_3_ perovskite, the XPS survey spectra of the MAPbI_3_@UiO-66 nanocomposites did not show the presence of any uncontrolled impurities. The absence of signals from the components of the hybrid MOFs was explained by its low concentration in the nanocomposite samples.

**Figure 10:**
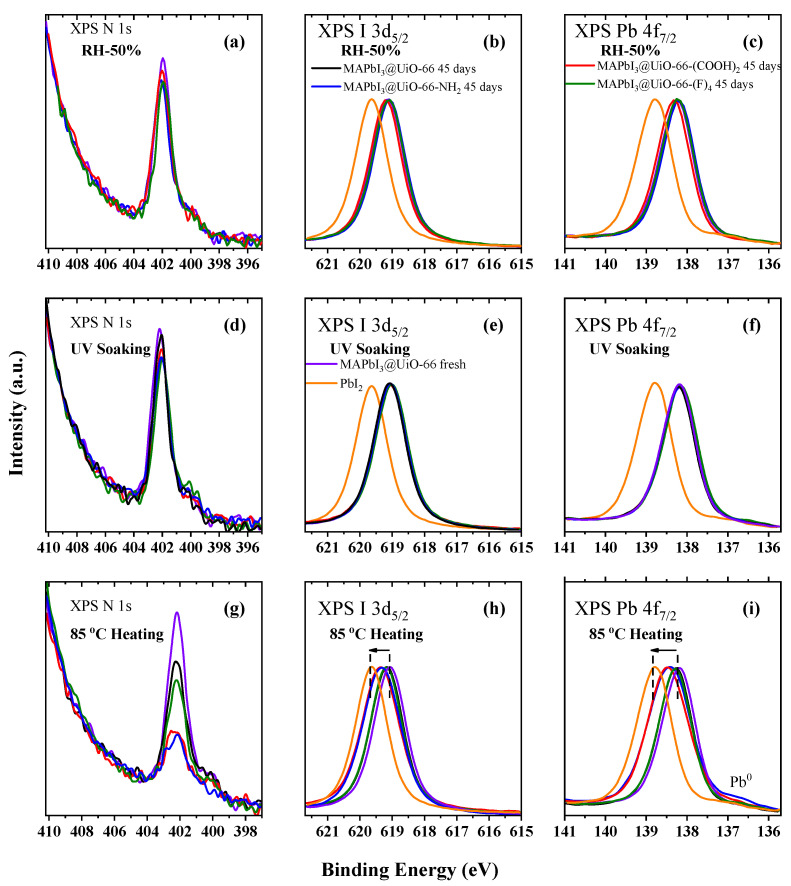
The XPS (**a**,**d**,**g**) N 1s, (**b**,**e**,**h**) I 3d, and (**c**,**f**,**i**) Pb 4f spectra of the MAPbI_3_@UiO-66 before and after (**a**–**c**) storage at RH 50%, (**d**–**f**) UV-light soaking, and (**g**–**i**) annealing at 85 °C for 45 days compared with the spectra of the PbI_2_ reference sample.

Figure 10a–c display the high-energy resolved XPS N 1s, I 3d, and Pb 4f spectra of the MAPbI_3_@UiO-66 nanocomposite samples before and after storage at RH 50% for 45 days. There were practically no changes in these spectra regardless of the type of the hybrid MOFs, revealing a good moisture stability. Moreover, these results showed that incorporation of the functional groups of COOH, NH_2_, and F into the organic ligands of UiO-66 did not reduce the hydrophobicity of the UiO-66 framework. Similar results were observed for the light soaking of these MAPbI_3_@UiO-66 nanocomposites. As shown in Figure 10d–f, the XPS N 1s, I 3d, and Pb 4f spectra did not show any significant changes during an exposure of 45 days regardless of the changes in the structure of the organic ligand. These results affirmed the high resistance of these MAPbI_3_@UiO-66 nanocomposites to UV-light irradiation. Finally, the last and most severe aging condition for the stability testing of these MAPbI_3_@UiO-66 nanocomposites to external influences was prolonged annealing at a high temperature of 85 °C. In this case (as with the results of the measurements of the XPS spectra shown in Figure 10g–i), the organic ligands in the UiO-66 and UiO-66-(F)_4_ were the most resistant to heat stress. Upon annealing of the MAPbI_3_@UiO-66 nanocomposites with the functional groups of COOH and NH_2_, a strong reduction in the relative intensity of the XPS N 1s-spectra was observed, and high-energy shifts toward the direction of the XPS spectra of the PbI_2_ compound were observed in the XPS I 3d and Pb 4f-spectra (Table 2). This indicated the partial decomposition of the organic cation and the PbI_2_ phase separation. In brief, we successfully analyzed a series of MAPbI_3_@UiO-66 nanocomposites by examining their compositional changes after aging with exposure to UV light, humidity, and a constant heat stress. Through comprehensively comparing the perovskite’s compositional changes after the aging conditions, correlated the stability difference with the structures of the hybrid MOFs. The improved stability toward RH 50% and UV light exposure was exerted by the blended MOFs regardless of the structures of the constituent organic ligands. The MAPbI_3_@UiO-66 and MAPbI_3_@UiO-66-(F)_4_ were shown to yield higher thermal stability than the MAPbI_3_@UiO-66-NH_2_ and MAPbI_3_@UiO-66-(COOH)_2_ as a result of their relatively higher affinity with the perovskite crystals.

## 4. Conclusions

It is no coincidence that the hybrid perovskites (2009) and metal–organic frameworks (1995) are mentioned among the most important discoveries in the field of materials for energy technologies [43]. The next step was to combine these two promising materials in “one bottle” and/or form nanocomposites [21,22,23,26,27]. The intensive research and determination of their possibilities in photovoltaics are just beginning, and in this regard, it was primarily of interest to experimentally determine their behaviors under external influences (irradiation, constant heat, and humidity), i.e., under conditions as close as possible to the operation of solar cells [44,45,46,47]. For these purposes, fundamental analyses of the XRD/SEM measurements helped to define the MOF-containing perovskite films with unaffected perovskite crystal growth, and the optical properties of UV/PL measurements showed the passivation effect of the MOFs and the luminescence variety with different aging conditions. To better understand the compositional difference within the aged samples, the present study used the method of XPS as an elemental and symmetry-selective probe to analyze the local atomic and electronic structure, which is especially important in the study of multicomponent systems. Using this method, we found that the MAPbI_3_@UiO-66 nanocomposites, even with a small amount of MOF, were sufficiently stable materials for photovoltaic applications when exposed to light, heat, and humidity. At the same time, the structural compatibility with the perovskite crystals and the incorporation of some additional functional groups, especially the fluorinated group, in MOF architecture design altered the affinity between MOF and perovskite. The UiO-66 and UiO-66-(F)_4_ MOFs demonstrated better stabilization of the perovskite interface, resulting in higher heat tolerance than the other two MOFs with the functional groups of COOH and NH_2_.

## Figures and Tables

**Table 1:** The positions of the Pb 4f_7/2_ and I 3d_5/2_ peaks of the MAPbI_3_ samples before and after various treatments (RH-50%, UV, and annealing at 85 °C).

Sample	Pb 4f_7/2_	I 3d_5/2_
PbI_2_	138.78	619.64
0 days	138.18	619.04
RH-50% 45 days	138.26	619.13
UV 45 days	138.23	619.08
85 °C 45 days	138.39	619.27

**Table 2:** Positions of the Pb 4f_7/2_ and I 3d_5/2_ peaks of the MAPbI_3_@MOF samples before and after annealing.

Sample	Pb 4f_7/2_	I 3d_5/2_
MAPbI_3_@UiO-66 85 °C	138.26	619.17
MAPbI_3_@UiO-66-NH_2_ 85 °C	138.41	619.33
MAPbI_3_@UiO-66-(COOH)_2_ 85 °C	138.49	619.36
MAPbI_3_@UiO-66-(F)_4_ 85 °C	138.28	619.18

## Data Availability

The data are contained within the article.
